# Electric Field Distribution in Bipolar Electrochemical Cells: Effects on the Wirefree Electrodeposition of Conducting Polymer Films

**DOI:** 10.1021/acs.analchem.4c04454

**Published:** 2024-12-19

**Authors:** Áine Brady, Robert J. Forster

**Affiliations:** † National Centre for Sensor Research, School of Chemical Sciences, Dublin City University, Dublin 9 D09 V209, Ireland; ‡ FutureNeuro, SFI Research Centre for Chronic and Rare Neurological Diseases, Dublin City University, Dublin 9 D09 V209, Ireland

## Abstract

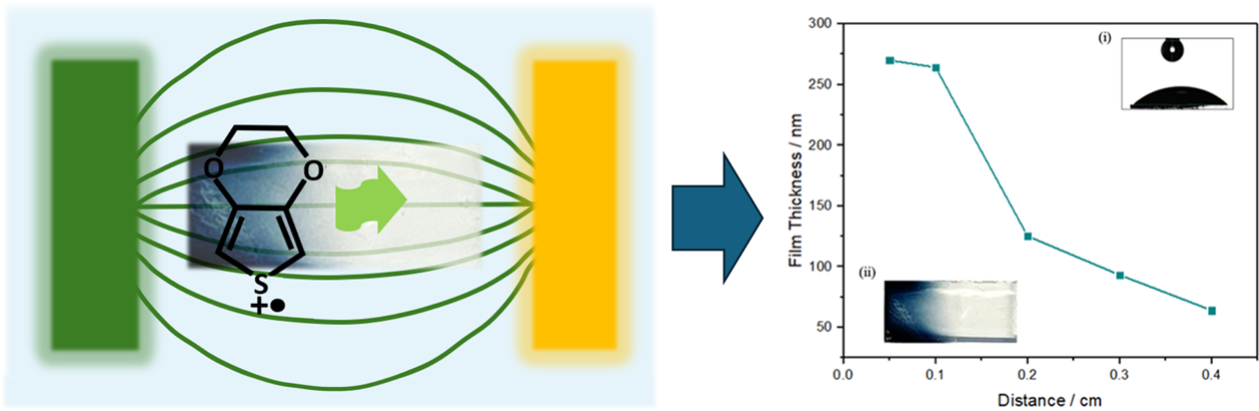

Wirefree, or bipolar electrochemistry, is advancing key fields, including (nano)materials, human health, and energy. Central to these applications is an understanding of the potential distribution induced in the bipolar electrode, BPE. Here, the impact of the electric field distribution is reported for the wirefree deposition of the conducting polymer, poly(3,4-ethylenedioxythiophene), PEDOT, in the absence of deliberately added electrolytes. PEDOT films with a gradient thickness are deposited, and the films formed at 10 V cm^–1^ for 20 min have an average film thickness of 350 nm. Significantly, the quantity of the polymer deposited increases proportionally to the deposition time up to approximately 20 min, suggesting that the presence of a thin PEDOT film does not change the interfacial potential distribution or driving force for heterogeneous electron transfer. For electric field strengths ≥5 V cm^–1^, PEDOT is deposited on regions of the BPE where the voltage is predicted to be insufficient to drive electropolymerization. This result demonstrates that local intensification of the field, e.g., at edges, and migration of the cationic radicals can significantly affect the electrodeposition profile. These results provide an enhanced understanding of the potential profiles for applications from multianalyte detection devices to wirefree electroceuticals.

## Introduction

Electrochemistry is central to contemporary analytical chemistry, by delivering highly sensitive and selective detection of targets relevant for medical, food, and environmental applications, as well as advanced (nano)materials with unique properties. It enjoys significant advantages over spectroscopy in terms of portability, miniaturization, and instrumental simplicity. However, unlike electromagnetic radiation that can be directed into a sample without a physical connection to an instrument, conventional electrochemistry requires the electrode to be physically connected (wired) to a potentiostat. Moreover, the potential of the working electrode in a traditional cell is uniform across its entire surface, meaning that the effects of different applied potentials must be investigated sequentially, e.g., using cyclic voltammetry. Also, in contrast to spectroscopy, conventional electrochemistry typically needs an electrolyte to be added to the sample which can change its properties. Wireless, or bipolar electrochemistry can address these issues by using an electric field that propagates through the solution, allowing the potential of the conducting working electrode to be controlled without a direct electrical connection.^1−3^ In its simplest incarnation, two driving or feeder electrodes are placed either side of a bipolar electrode (BPE) and connected to a power supply, generating an electric field.^2,4−6^ The solution phase potential created by the field can then be used to wirelessly induce local potentials within the BPE, whose magnitude depends on the field strength, BPE length, the electrolyte concentration, and other less well-documented factors such as the number of feeder electrodes and their relative orientation.^7,8^ Similar to a conventional cell, the potentials induced in the BPE can drive reduction and oxidation processes, but the specific reactions can change across the BPE surface as the induced potential depends on the position along the BPE.^9^ Moreover, the system can be designed so that current flows predominantly through the BPE, enabling wireless electrochemistry to be performed in the absence of a deliberately added electrolyte. Thus, it directly addresses the major shortcomings of electrochemistry compared to spectroscopy while maintaining its key advantages.

In this contribution, we report on the impact of the electric field strength on the electrodeposition of the conducting polymer, poly(3,4-ethylenedioxythiophene), (PEDOT) in the presence of Milli-Q water. Conducting polymers (CPs) are appealing materials because of their high conductivity, flexibility, and stability.^10−12^ The redox potential, conductivity, hydrophilicity, and surface charge of these materials can be systematically varied, allowing them to powerfully enable electrochemical communication with cells and tissue, potentially leading to wirefree electroceutical devices.^1^ For example, cell differentiation and growth as well as cancer cell destruction could be driven wirelessly.^1,13,14^ Furthermore, CPs can also be used as drug delivery vehicles since they can release Active Pharmaceutical Ingredients (APIs) upon electrochemical stimulation.^15−17^ In conjunction with bipolar electrochemistry, “Wirefree” drug delivery systems (DDS) could revolutionize the treatment of chronic diseases.^2,18,19^ Beyond these 4D properties, the *in situ* polymerization of CP films without the requirement of a physical connection to a working electrode opens up many opportunities for innovative applications. For example, a biocompatible monomer and stem cell suspension could be injected around a metallic stent where an electric field could initiate polymerization and promote cell differentiation and growth for regenerative medical purposes. Additionally, on-demand drug release could be designed to recharge in situ, minimizing the need for surgical implantation.

Elegant experiments have been performed to model and map the potential distribution, notably by Inagi using electrochemiluminescence.^20^ Additionally, experimental and simulation work using Finite Element Method (FEM) modeling has also been performed,^8^ however, the impact of the electric field strength and distribution, the magnitude of the potentials induced in the BPE, and the effects of electrophoretic mass transport, e.g., cation radicals, merit further attention for the electrodeposition of CPs. A key goal is to elucidate the applicability of the conventional linear potential decay model for the electrodeposition of CPs in the absence of deliberately added electrolyte. This contribution reports on the impact of the electric field strength on the location of electrodeposition and the nature of the polymer deposited. Significantly, we demonstrate that well-defined films can be formed using bipolar electrochemistry without intentionally added electrolyte, potentially giving new insights into the relative importance of electronic and ionic conductivity in these materials. Here, the properties of PEDOT films prepared using bipolar electrochemistry in Milli-Q water are compared with those produced conventionally in aqueous 0.1 M LiClO_4_, in terms of film thicknesses, electronic structure, homogeneity, morphology, and surface free energy. The results give a promising insight into the possibilities of conducting polymers in bipolar electrochemistry for application in cell stimulation,^2^ closed-loop drug delivery systems,^21^ and areas such as non-invasive monitoring, where the wireless capability facilitates the development of *in vivo* sensors.

## Experimental Section

### Materials

All materials used throughout this experimentation were of reagent grade, obtained from Merck, and used as received. Purified water was used throughout and had a resistivity of 18.2 MΩ cm.

### Bipolar Setup

The bipolar electrochemical cell was designed in house using FreeCAD and printed using Acrylonitrile Butadiene Styrene (ABS) Ultimaker filament with an Ultimaker S5 3D printer (Figure S1). In this cell, two, L:2 cm × W:1 cm × D:2 mm titanium feeder electrodes sit 3 cm apart within two slots. A L:2 ± 0.2 cm × W:1 ± 0.2 cm bipolar Fluorine-doped Tin Oxide (FTO) electrode sits centrally between the two feeder electrodes within a slot to ensure reproducible alignment. Two wells on either side of the FTO bipolar electrode allow for easy removal using tweezers without damaging the polymer film. The FreeCAD stl file was exported and Cura software was used to adjust specific parameters (resolution, infill density, temperature) for 3D printing. The cell was printed with ABS filament using “normal” resolution, 0.15 mm, with an infill density of 20%, and a temperature of 230 °C. An EA PS 5200–02A power supply was used to supply the desired voltage to the feeder electrodes. The FTO bipolar electrodes were sonicated in alcohol and dried using nitrogen prior to use. The titanium feeder electrodes were cleaned using 1600 grit sandpaper (≈10 μm particle size) between experiments. For bipolar polymerization, the cell contained 3 mL of 10 mM EDOT dissolved in Milli-Q water. The voltage induced across the BPE was calculated using eq 1:^9^
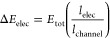
1where *l*_channel_ is the distance between driving electrodes (constant at 3 cm), *l*_elec_ is the length of the BPE (2 cm), and *E*_tot_ is the voltage difference applied to the pair of feeders. In the following description, the negative feeder is considered to be on the left-hand side so that the positive pole of the BPE, where oxidative polymerization of EDOT can occur, also lies on the left.

Eq 1 is currently the most widely used method of estimating the voltage induced across the BPE and has been used here to enable different experiments to be compared.

### Conventional Three-Electrode Cell

Cyclic voltammetry was performed using an AutoLab (Metrohm) systems potentiostat. The anodic pole of the FTO working electrode (2 ± 0.2 cm × 1 ± 0.2 cm), was masked with Teflon tape to give a geometric working surface area of 1 cm^2^. The reference electrode used was Ag/AgCl (saturated KCl), and the counter electrode was a coiled platinum wire (≈3.6 cm^2^). For conventional polymerization, galvanostatic polymerization was performed using a current of 200 μA cm^–2^. Cyclic voltammetry of bipolar films, prepared in Milli-Q water, and those produced galvanostatically, were performed in aqueous 0.1 M LiClO_4_ using scan rates ranging from 5 to 100 mVs^–1^. The film thickness was calculated assuming a volumetric capacitance for PEDOT of 100 F cm^–3^.^22^

### Profilometry

Profilometry was performed on the Bruker DektakXT profilometer. PEDOT films were grown using bipolar polymerization at 10 V cm^–1^ for 20 min using 10 mM EDOT in Milli-Q water. Since the field changes from left to right but is uniform from top to bottom, the upper half of the polymer film was removed to allow the film thickness to be determined at different positions along the BPE. The stylus was moved from the bare FTO side onto the polymer film. Profilometry was performed on two independent samples at distances of 0.05, 0.1, 0.2, 0.3, and 0.4 cm from the edge of the positive BPE pole.

### UV–Vis Spectroscopy

UV–vis spectroscopy was performed using the Agilent Cary UV–Vis spectrometer. Samples included a conventional PEDOT/ClO_4_ film, as well as a bipolar PEDOT film which was polymerized at 10 V cm^–1^ for 20 min on FTO. The PEDOT films on FTO were loaded into an in-house 3D printed sample holder within the spectrometer which allowed the region where the spectrum is recorded to be controlled while maintaining the sample vertical.

### Microscopy

A Nikon eclipse ME600 microscope was used to visualize the polymerization profile on the BPE. This was achieved by measuring the distance from the positive edge of the BPE to the location where there is no polymer film deposited. The microscope images were captured using a camera inserted into the microscope and were displayed using S-EYE software. The percentage of the surface covered was calculated by dividing the measured polymerization distance by the length of the BPE.

### Scanning Electron Microscopy

A Jeol JSM-IT 100 InTouchScope electron microscope was used to image the PEDOT films on FTO using an accelerating voltage of 2.0 kV and a secondary electron detector.

### Surface Free Energy

Contact angle measurements were performed using an FTA200 Dynamic Contact Angle analyzer. A single droplet of deionized water was deposited on the bare FTO, and PEDOT films prepared using bipolar and galvanostatic approaches, and the resulting contact angle was measured. PEDOT films formed using the bipolar setup were polymerized in a 10 mM EDOT in Milli-Q water at 10 V cm^–1^ for 20 min. Conventional polymerization was performed by applying 200 μA cm^–2^ for 300 s in 10 mM EDOT 0.1 M LiClO_4_ solution as this deposited a similar quantity of PEDOT as the 10 V cm^–1^ 20 min bipolar experiments.

## Results and Discussion

### Surface Characterization

**Figure 1:**
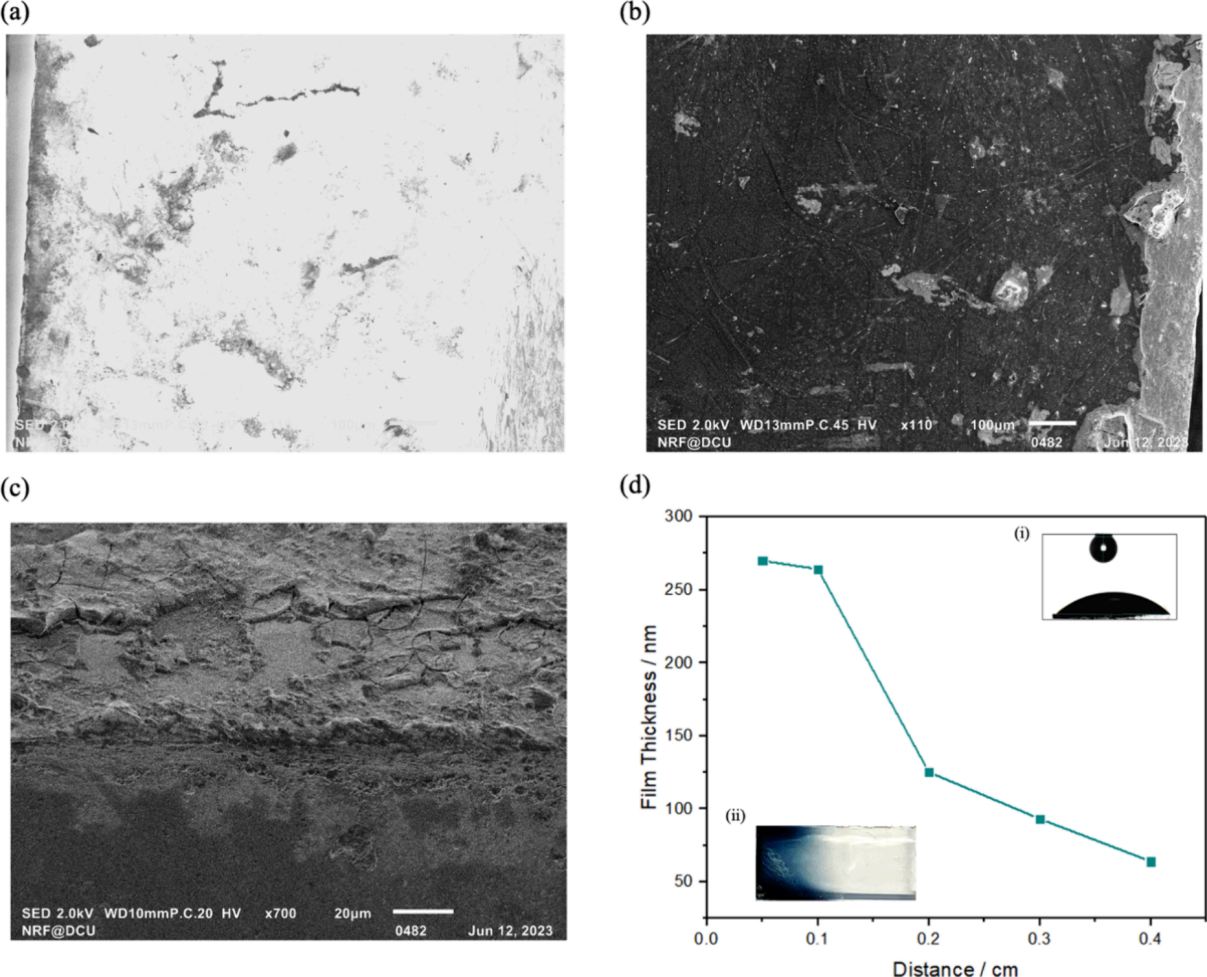
SEM images of the BPE after polymerization at 10 V cm^–1^ for 20 min in 10 mM EDOT in Milli-Q water. (a) Negative pole. (b) Positive pole. (c) Long edge perpendicular to feeder (2 cm side). (d) Dependence of the film thickness with increasing distance from edge of the positive BPE pole toward the center of the BPE using profilometry (*n* = 2) Insets: (i) Contact angle measurement and (ii) photograph of the bipolar film formed at 10 V cm^–1^ after 20 min.

SEM provides useful information regarding the morphology (e.g., porosity, roughness, patterning) and structural characteristics of polymer films. Here, SEM was used to determine where PEDOT deposits with a view to correlating that with the expected potential distribution across the BPE. Figure 1a shows the negative pole of the BPE after polymerization at 10 V cm^–1^ for 20 min in a 10 mM EDOT Milli-Q water solution. Figure 1b shows the SEM image of the positive pole (LHS) of the BPE. In contrast to the negative pole, significant film formation is observed that has a rough, irregular morphology. The formation and extent of the film depends on the voltage applied to the feeder electrodes/electric field strength with film formation only being observed for fields greater than approximately 1.7 V cm^–1^. Assuming a linear decay through solution, this field could induce a maximum voltage of ±1.7 V at the tips of the BPE. In a conventional three-electrode cell, EDOT oxidizes at approximately +0.85 V albeit in the presence of 0.1 M LiClO_4_ as supporting electrolyte.^23^

The surface of the polymer film has irregular ridges, and the layer is thicker around all three edges of the BPE. These differences may arise from a more intense electric field due to field focusing or migrational mass transport/stirring effects. Figure 1c shows a side view of the PEDOT film (positive pole of the BPE), revealing the rough, layered film on the FTO substrate. Polymer cracking is visible, which is consistent with drying induced stress.^24^ The rough texture of the film could promote stable cell adhesion, which would broaden the uses of this substrate for applications such as cell stimulation.^2,25^

The potential induced across the BPE varies along its length, and with the rate of heterogeneous electron transfer affecting the deposition rate, the potential distribution should be reflected in the resulting film thickness distribution. Therefore, stylus profilometry was performed to gain insight into the film thickness along the BPE (Figure 1d). The profilometry data indicates that the film is thicker at the edge of the positive pole of the BPE and becomes thinner moving toward the center of the BPE. These results are consistent with the position dependent induced voltage influencing the rate of polymer deposition, i.e., the more positive the induced voltage the more polymer is deposited per unit time. Significantly, there are substantial edge effects, not only as expected at the edge parallel to the feeder, but the film is also thicker at both edges of the BPE that are perpendicular to the feeder electrodes. This gives rise to a “U-shaped” deposition (Figure 1d,i). This behavior could arise because the field is not uniform across the BPE (along the axis parallel to the feeder) due to the similar widths of feeders and BPE, distortions in the electric field due to the current flow through the BPE, or enhanced mass transfer to the edges due to edge effects.

The contact angle was measured to probe the hydrophobic or hydrophilic nature of the bipolar (BP) PEDOT films, (Figure 1d,ii). The different potentials induced along the length of the BPE are also likely to result in different redox compositions within the CP, but once the bipolar stimulation is removed, the redox composition is expected to become uniform. Significantly, the contact angles differ along the length of the BPE, with the PEDOT film at the positive BPE pole having a more hydrophilic nature, (where the film is thicker due to a faster deposition rate) with an average contact angle of 49° ± 3° (two independent samples), than the film toward the center of the BPE with an average contact angle of 55° ± 4° (two independent samples). This behavior could arise from differences in film structure due to the relatively higher deposition at the BPE tip. It is also important to note that the increase in hydrophilicity at the BPE anode could also be influenced by overoxidation as the experimentally calculated expected voltages exceed 1.5 V.^26^ Any differences in redox composition are considered later using UV–vis spectroscopy. The observation that the wirefree deposited PEDOT films are hydrophilic could make them a potentially useful platform for the adhesion of biological cells and biosensors.^25,27^

### UV–Vis Spectroscopy

UV–Vis spectroscopy can provide useful insights into the chemical composition and band gaps of electronically conducting polymers, both of which can be influenced by the deposition conditions, e.g., applied/induced potential, deposition rate, electrolyte etc.^28,29^ Moreover, UV–Vis can be used to give an insight into the oxidation and doping states of the material. For example, doped PEDOT shows conductivity levels of the order of 5000 S/cm, and 0.1 S/cm in the undoped form which is reflected in the UV–Vis spectrum.^30,31^ Understanding the extent of doping is especially important here since polymerization occurs in “electrolyte free” media, perhaps allowing the relative importance of electronic and ionic conductivity to be uniquely investigated. While the experiments were carried out in Milli-Q water without any added electrolyte, it is important to consider the possibility that wireless electrochemistry generates ions. Independent measurements of the solution conductivity reveal that the conductivities for the 10 mM EDOT solution is 4.6 ± 0.5 μS cm^–1^ which increases to 6.2 ± 0.6 μS cm^–1^ after 20 min of bipolar electrochemistry where 30 V is applied across the pair of feeder electrodes. The postelectrolysis conductivity corresponds to a concentration of approximately 75 μM of a univalent electrolyte, i.e., applying the electric field does not generate significant quantities of ions. To investigate the possibility of hydroxide ions doping the PEDOT films, the amount of moles of monomer on the electrode surface was calculated to be 2.46 × 10^–7^ moles of EDOT, assuming a volumetric capacitance of 100 Fcm^–3^.^22^ Assuming a hydroxide solution concentration of 3 × 10^–10^ moles (in 3 mL), and a polaron approximately every four repeating units of the polymer,^32^ the solution has a sufficient concentration of hydroxide ions to have a low-doped PEDOT film.

**Figure 2:**
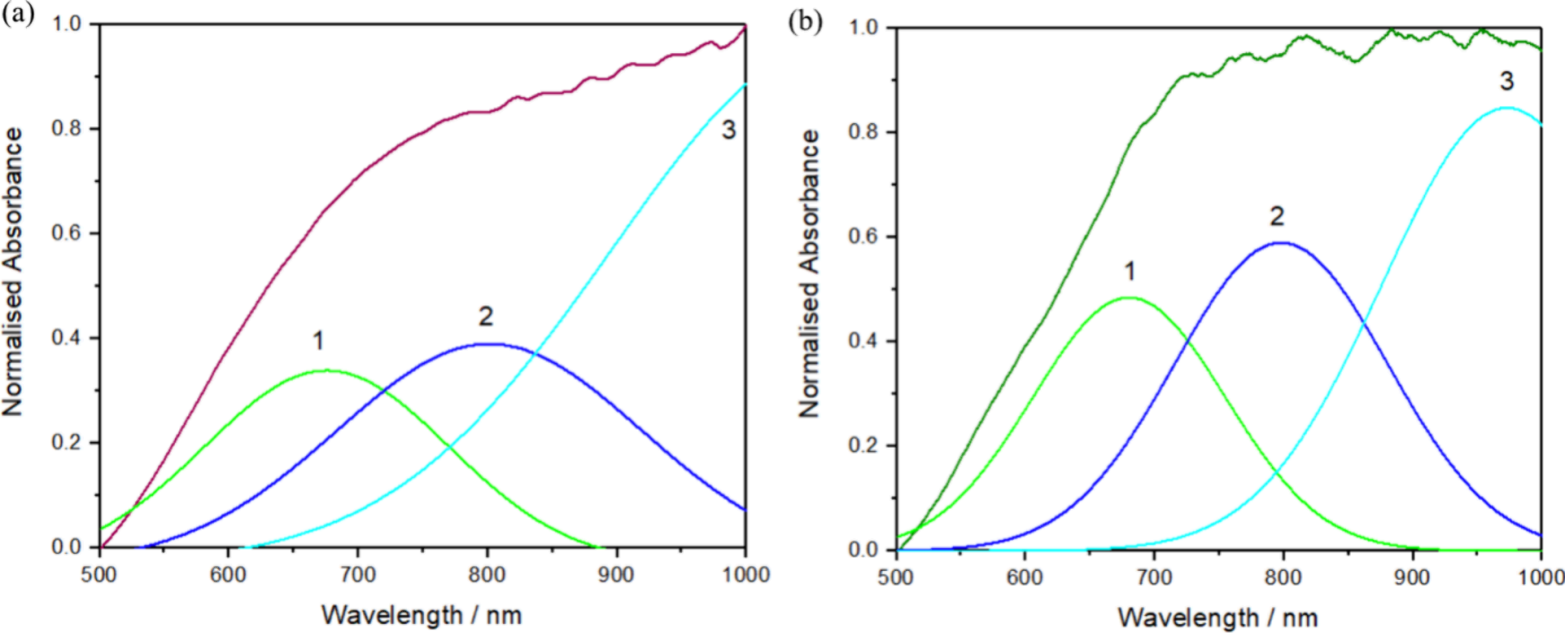
Normalized absorbance of 400 nm–1000 nm wavelength light through (a) conventionally deposited PEDOT/ClO_4_ on FTO (1 cm × 1 cm) by applying 200 μA cm^–2^ for 300 s in 10 mM EDOT and 0.1 M LiClO_4_ (purple) and (b) bipolar-deposited PEDOT on FTO polymerized at 10 V cm^–1^ for 20 min in 10 mM EDOT in Milli-Q water (green). The curves 1, 2, and 3 represent the Gaussian curves for the best fit to the experimental spectra.

Figure 2a shows the UV–Vis spectrum for a PEDOT film that was electrodeposited in 10 mM EDOT in 0.1 M LiClO_4_ at a current of 200 μA cm^–2^ for 300 s. Figure 2b shows the UV–Vis spectrum for a PEDOT film deposited using a 2 cm long FTO BPE with an electric field of 10 V cm^–1^ for 20 min in Milli-Q water.

Figure 2 shows that the material deposited using bipolar and conventional approaches are similar to both absorbing strongly from 650 to 1000 nm which is consistent with polaronic states being present in both films.^33^ This is an interesting observation as the BP polymerization of EDOT was performed in Milli-Q water, with no intentionally added electrolyte, perhaps leading to a low-doped film. Gaussian deconvolution was performed on both spectra to determine the wavelengths of the individual electronic transitions. Both spectra are adequately fitted using a minimum of three Gaussian peaks. The peak at 680 nm for both films corresponds to the transition of a lower polaronic band to a higher polaronic band while the second peak which occurs at approximately 830 and 800 nm for PEDOT/ClO_4_ and BP films respectively, also corresponds to polaronic transitions. The third transition which occurs at 1000 nm is a result of both polaron and potentially bipolaron absorption.^34,35^ These results suggest that both the BP and conventional films possess similar electronic states and possibly similar doping levels. This is a significant result due to the absence of electrolyte during the bipolar polymerization process.

### Conventional Polymerization

PEDOT films were also produced using galvanostatic deposition in a three-electrode cell at a current density of 200 μA cm^–2^ for 100, 200, 300, and 600 s in LiClO_4_. The conventional PEDOT films are intentionally doped with ClO_4_^–^. The galvanostatic deposition times were chosen to deposit a similar quantity of PEDOT as that deposited in the wireless experiments. The film capacitance depends on the quantity of PEDOT deposited and, to a lesser extent, its thickness and composition/physicochemical properties.

**Figure 3:**
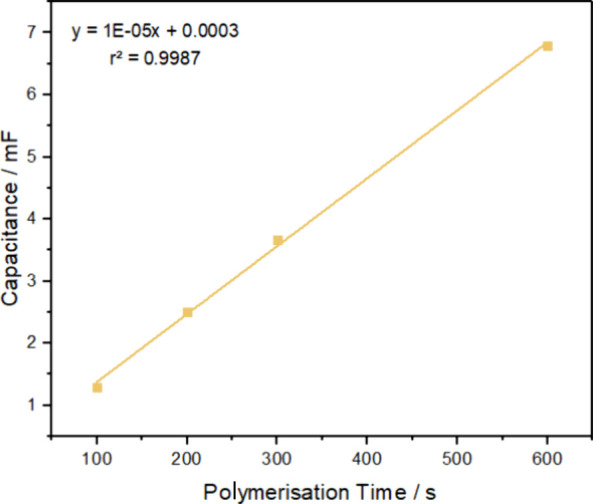
Conventional film capacitance obtained by scan rate study of the PEDOT/ClO_4_ film in 0.1 M LiClO_4_ from 5 to 100 mV/s versus polymerization time. Conventional polymerization was performed at 200 μA for 100, 200, 300, and 600 s in 10 mM EDOT 0.1 M LiClO_4_. Error bars are comparable to the size of the symbols.

Figure 3 shows the dependence of film capacitance on time, measured from the current observed in cyclic voltammetry from 5 to 100 mVs^–1^ at 0.2 V where the current is dominated by film charging and no faradaic processes occur. The capacitance increases in direct proportionality to the deposition time suggesting that the quantity of PEDOT deposited depends directly on the deposition time. A direct comparison between the conventional and bipolar PEDOT films appears reasonable since the UV–Vis spectra and film structures of the BP and conventional films are similar (small cauliflower like aggregates),^36^ suggesting that their intrinsic capacitances should be comparable. The BP film polymerized at a field strength of 10 V cm^–1^ for 20 min (3.5 mF) has a similar capacitance to that found for approximately 300 s (3.7 mF) of galvanostatic polymerization at 200 μA in a conventional three-electrode cell. The film thickness was calculated by assuming a volumetric capacitance of 100 F cm^–3^,^22^ and the BP film polymerized at 10 V cm^–1^ (30 V) for 20 min has an average film thickness of 350 nm (disregarding the gradient deposition) (2.46 × 10^–7^ mol) which corresponds to approximately 300 s of galvanostatic polymerization at 200 μA cm^–2^ in a conventional three-electrode cell producing a film thickness of 370 nm (2.60 × 10^–7^ mol). In conclusion, the combination of the UV–Vis, profilometry, and cyclic voltammetry data suggest that the material deposited in the wireless process is PEDOT and that its structure and capacitance are similar to that found using galvanostatic deposition. Strikingly, despite the absence of deliberately added electrolyte, the band gap in the BPE experiments is indistinguishable from conventional PEDOT on the basis of the UV–Visible spectra.

### Influence of Deposition Time on BP PEDOT Film Formation

**Figure 4:**
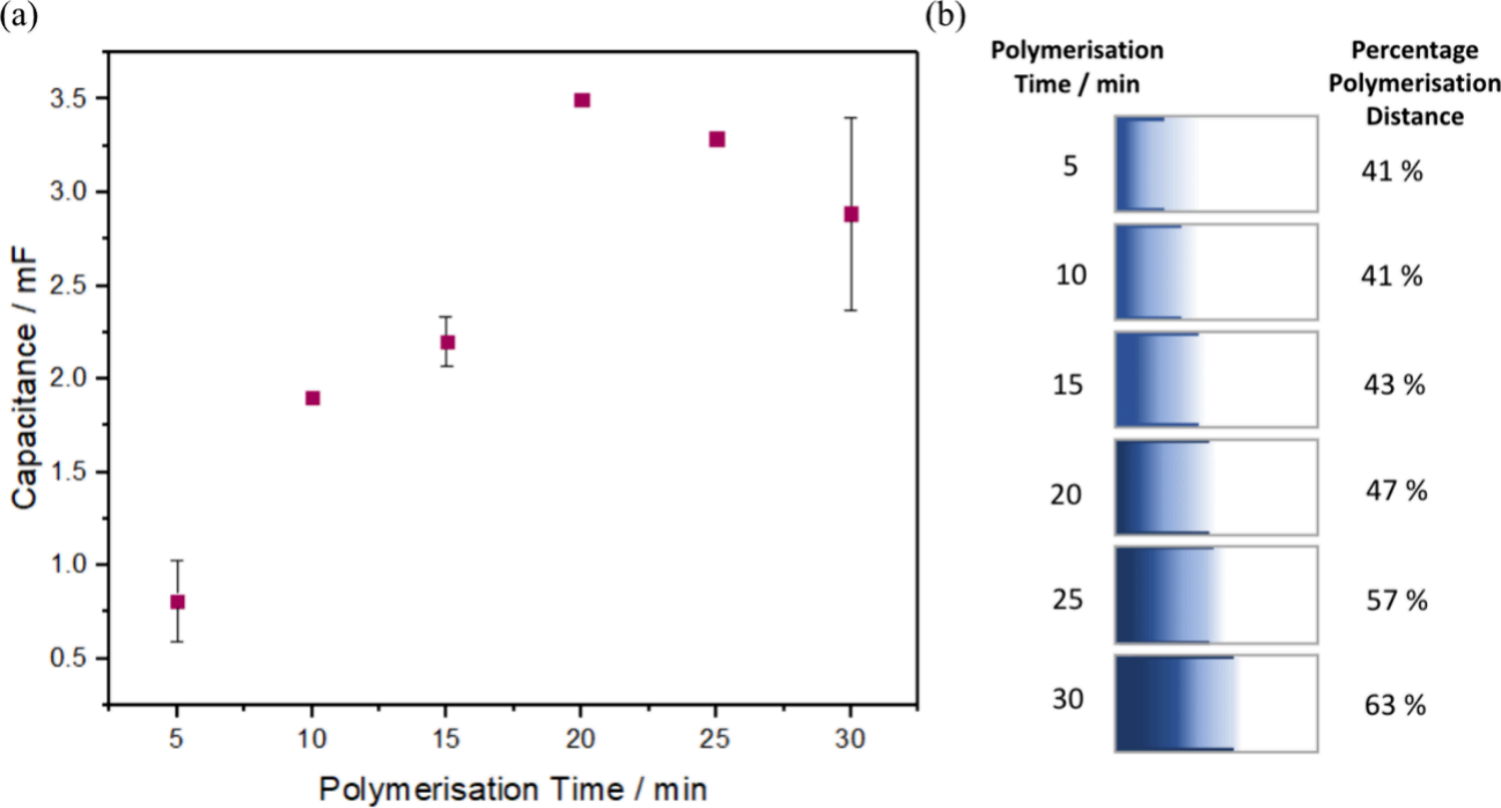
Influence of time on BP polymerization. (a) Capacitance of deposited PEDOT at a constant 30 V (10 V cm^–1^) for 5–30 min in a 10 mM EDOT Milli-Q water solution. (b) Percentage polymerization distance across the length of the BPE. Polymerization was performed at a constant 30 V (10 V cm^–1^) for varying times of 5–30 min. The polymerization distance was calculated as outlined in the Experimental Section. Where error bars are not visible, they are comparable to the size of the data points.

The potential across the BPE is generally assumed to decrease linearly from the edge of the BPE facing the feeder electrode toward 0.000 V at its center. Thus, only a certain fraction of the BPE has a sufficiently positive potential to drive PEDOT formation. A longer deposition time is expected to increase the amount of PEDOT deposited, however, it should not affect the area over which deposition occurs due to the potential profiles. The effect of deposition time on wireless deposition was investigated using a field strength of 10 V cm^–1^ for 5, 10, 15, 20, 25, and 30 min. The capacitance of the bare FTO electrode was less than 0.05% of that found for the PEDOT coated electrode but was subtracted from the value found for the functionalized electrode and the capacitance values in Figure 4 correspond to the quantity of deposited PEDOT. Figure 4a shows that the capacitance initially increases approximately linearly with increasing deposition time reflecting an increasing amount of PEDOT deposited before becoming independent of time after approximately 20 min. The observation of a limiting PEDOT capacitance after 20 min polymerization time may be due to slow homogeneous charge transport through the conducting polymer, thus limiting the rate of EDOT oxidation.^37^

Perhaps the most significant observation of Figure 4 is the time dependence of the length of area undergoing active PEDOT deposition. The length of the polymer band deposited was measured using optical microscopy and the fraction of the BPE length where PEDOT deposition is observed is given in Figure 4b. Significantly, Figure 4b indicates that the widely used linear decay of the electric field/induced voltage model does not fully explain the results obtained.^9,38^ That model predicts a zero potential in the center of the BPE with the rest of the BPE having an incrementally more positive or negative potential as one moves toward the tips of the BPE. Also, considering that the electric field (10 V cm^–1^), and the electrode length are both constant, the distance of the deposition region should not change over time. The experimental behavior suggests that factors such as current flow, diffusion, electrophoretic effects, and stirring may play a role in determining the polymerization distance, e.g., due to migration of the cation radicals in the electric field. First, the flow of current through the BPE can impact the electric field distribution and therefore influence the transport and heterogeneous electron transfer processes as well as the potential profiles across the BPE.^39,40^ Electrophoretic mass transport^41^ could play a significant role in the movement of charged cation radicals from the positive BPE pole to the negative BPE pole (Figure S2), increasing the polymerization distance as the charged oligomers would move in the direction toward the negative BP pole (Figure 6ii) before the increasing molecular weight increase to the point where they become insoluble and deposit.

### Influence of the Feeder Voltage on the Potential Profile

**Figure 5:**
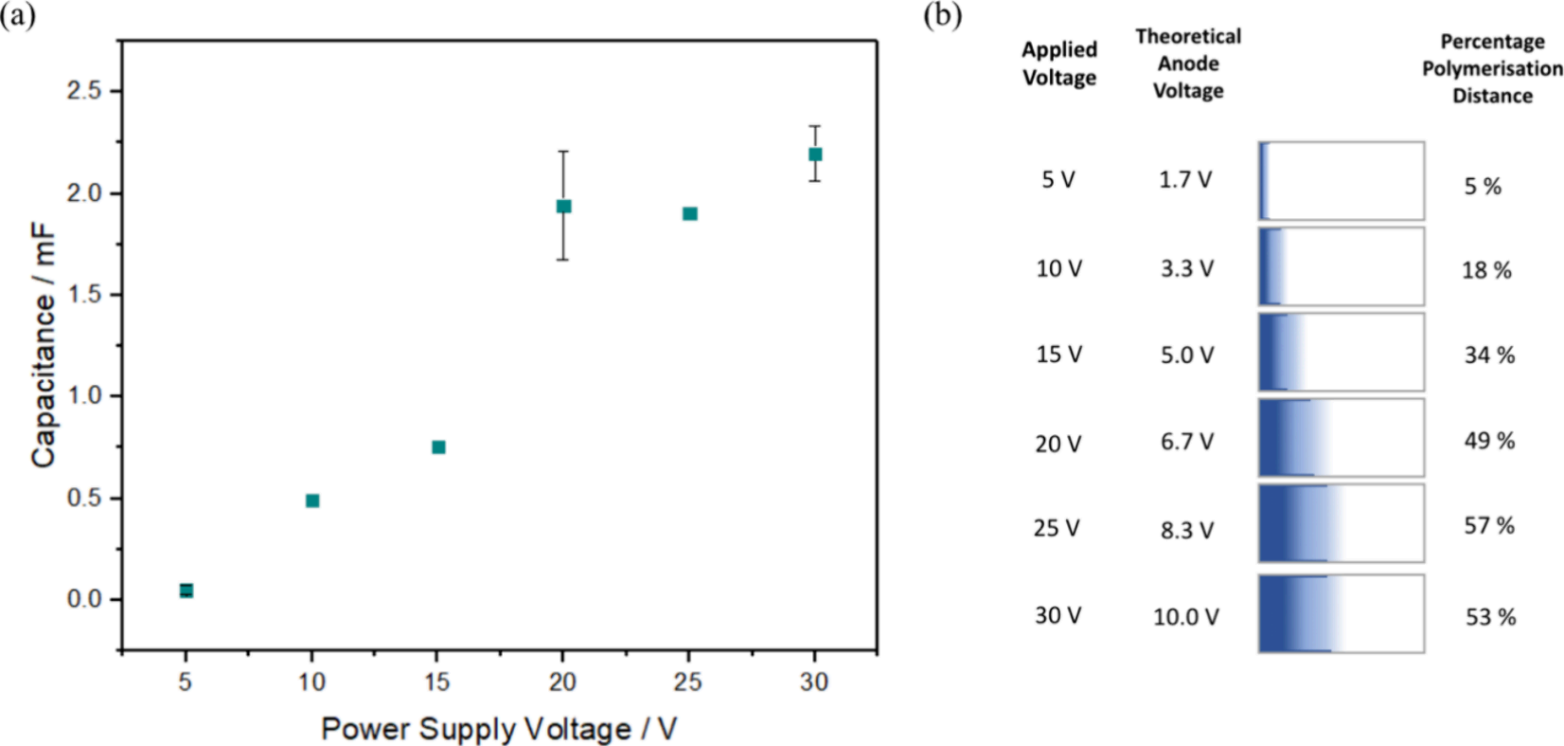
Influence of the voltage applied to the feeder electrodes on BP polymerization. (a) Dependence of the quantity (capacitance) of PEDOT deposited on the BPE as the voltage dropped across the two feeder electrodes is varied. The deposition time is 15 min. (b) Diagram of the bipolar-polymerized PEDOT on the FTO surface at varying voltages for a constant time of 15 min. BP polymerization was performed in 10 mM EDOT Milli-Q water solution from 5 to 30 V. Where error bars are not visible, they are comparable to the size of the data points.

The effects of voltage on the wireless deposition of PEDOT was investigated by applying voltages of 5, 10, 15, 20, 25, and 30 V to the bipolar system while keeping the deposition time constant at 15 min. At very high induced potentials, the overpotential for EDOT oxidation could become so large that the Marcus inverted region effects cause the rate of heterogeneous electron transfer to become independent of the induced potential. In the absence of this effect, the conventional linear decay model predicts that the area where PEDOT formation can proceed should depend directly on the voltage dropped across the pair of feeder electrodes, causing a proportional increase in the quantity of PEDOT deposited. The quantity of PEDOT deposited was determined from the film capacitance using the scan rate, υ, dependent (5 ≤ υ ≤ 100 mV s^–1^) current at 0.2 V where the polymer modified electrode was the working electrode in a conventional three-electrode cell. Figure 5 shows that the capacitance increases with increasing electric field strength which is consistent with the rate of polymer deposition being influenced by the rate of heterogeneous electron transfer that depends on the voltage induced in the BPE. However; this relationship is not proportional, i.e., doubling the voltage increases the quantity of PEDOT deposited more than expected unlike conventional deposition (Figure 3).^42^

### Effect of Electric Field Strength on the PEDOT Deposition Area

**Figure 6:**
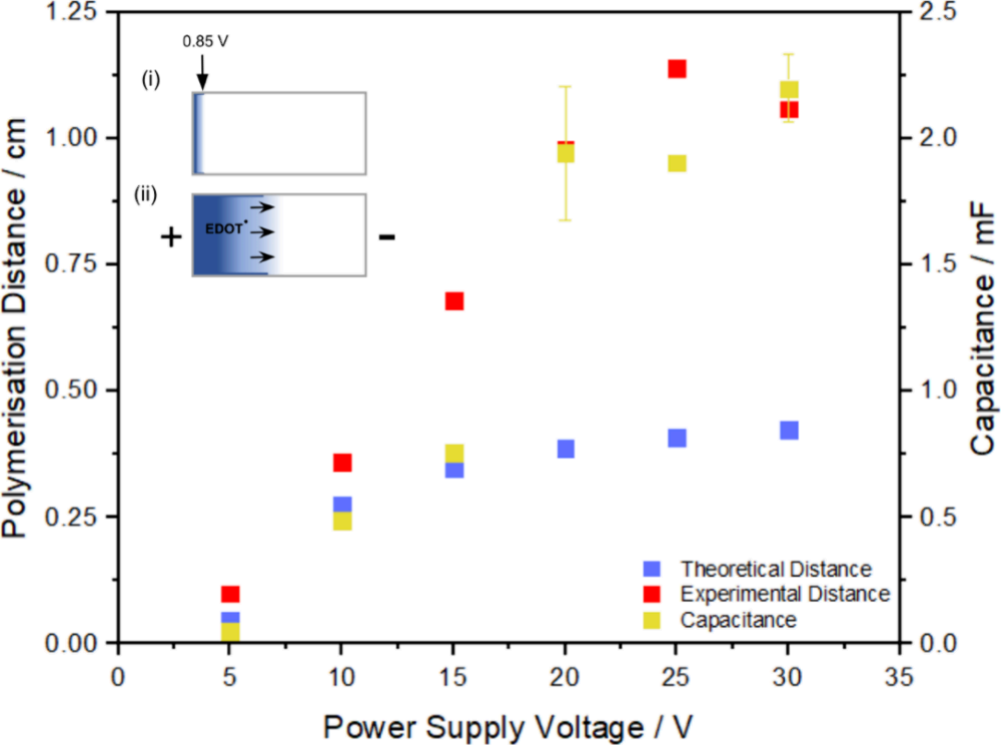
Left axis: Experimental polymerization distances (red) and theoretical polymerization distances calculated using the 5 V sample (blue) at varying voltages. Right axis: Wireless PEDOT film capacitances that were polymerized at varying voltages. Capacitance values were determined as described in the previous section. Insets: (i) Graphic of the 5 V sample where the voltage at the location where polymerization starts is assumed to be +0.85 V and (ii) graphical representation of the possible electrophoretic movement of cationic EDOT radicals at high field strengths.

In order to further investigate the validity of the linear potential decay model and the potential distribution equation (eq 1), the expected polymerization distances were calculated. Inset (i) of Figure 6 shows the result obtained for a sample polymerized at 5 V for 15 min (5 V sample) in a 10 mM EDOT in Milli-Q water solution. Based on conventional electrode measurements, polymerization requires an induced potential more positive than +0.85 V, and the polymerization is expected to be controlled by the potential dependent rate of electron transfer. Considering the PEDOT polymerization distance of 0.1 cm for the 5 V sample, the electric field strength was calculated by assuming the start of the PEDOT film as +0.85 V, as shown in Figure 6i. Since the center of the bipolar electrode must be 0.000 V, every 0.1 cm equates to 0.094 V. This then results in the BPE anode experiencing 0.94 V cm^–1^ (electrode length of 2 cm) compared to the expected value of 1.7 V cm^–1^ (5 V dropped across the 3 cm cell) as per eq 1. At this low field strength, factors such as the migration of EDOT radicals toward the negative pole of the BPE are expected to have minimal influence on the polymer deposition region. This value, 0.94 V cm^–1^, was then used to theoretically calculate the expected polymerization distances (in the absence of migration) at different power supply voltages, which were compared with experimental capacitances and polymerization distances (Figure 6).

There are several important observations connected to Figure 6. First, for low feeder voltages (5 and 10 V) the predicted polymerization distances based on the absence of migration (blue) and the experimentally observed distances (red) are closely correlated, suggesting that the linear potential distribution model is appropriate for low field strengths. However, at 15 V there is a substantial deviation between the theoretical and experimental distances, with the experimental polymerization distance being approximately twice that of the theoretically calculated distance. This extended polymerization distance could arise due to migration of the cation radicals toward the negative pole of the bipolar electrode. This movement is likely as the field strengths that are calculated at higher voltages are similar to the 5–8 V cm^–1^ electric field strength that would typically be used for lower field electrophoresis.^43^Figure 6 shows the dependence of the film capacitance on the feeder voltage. Significantly, the capacitances increase approximately linearly with increasing feeder voltage up to 20 V before becoming independent. This result suggests that at higher electric field strengths *both* the deposition area and the quantity of PEDOT deposited increase. This result is not predicted by the standard linear decay of the potential across the BPE. A second important observation is that for feeder voltages above 20 V, PEDOT deposition is observed beyond the center of the bipolar electrode which the standard model considers to be at 0 V, which is insufficient to drive oxidation of the EDOT monomer. Together, these data indicate deficiencies in the standard linear decay model, at least under the current conditions.

**Figure 7:**
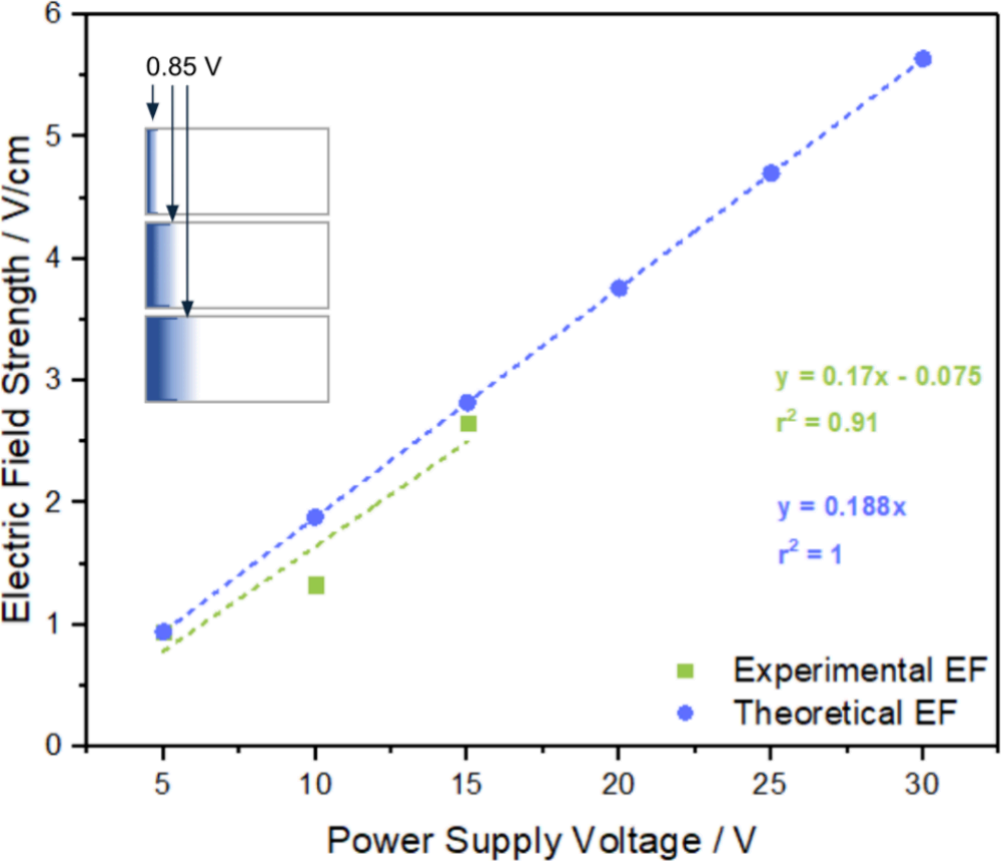
Theoretical electric field strength calculated based on the 5 V sample (blue) and experimental electric field strength calculated based on the initial polymerization band being +0.85 V. Inset: Graphical representation of the 5, 10, and 15 V samples showing where 0.85 V was extrapolated.

The theoretically calculated electric field strength based on the 5 V sample (blue) was then compared to the experimentally calculated electric field of the first three samples (5, 10, and 15 V) based on polymerization commencing at +0.85 V (green) (Figure 7 inset). Figure 7 shows that the calculated (blue) and experimental (green) electric field strengths depend on the feeder potential in a similar way for voltages up to 15 V, suggesting transport effects such as migration, stirring, or electrophoretic effects do not contribute significantly at these relatively lower field strengths.

## Conclusions

This work demonstrates that high quality PEDOT films can be electrodeposited wirelessly even in the absence of deliberately added electrolyte. The deposition pattern provides insights into the potential profile across the bipolar electrode and highlights the important role that processes, such as migration, can play when the field strength is relatively high (>5 V cm^–1^). Significantly, the findings suggest that the widely used linear decay of the electric field/induced voltage model does not fully explain the electrodeposition of PEDOT at higher electric field strengths. Under these conditions, PEDOT deposition can occur in regions where the induced potential is insufficient to drive monomer oxidation. The ability to use the electric field to extend the deposition region through active transport effects opens new possibilities in device fabrication, e.g., interconnect formation in low-cost, all-plastic electronics. Significantly, despite the fact that applying the electric field does not generate a significant concentration of ions, UV–Vis spectra of the PEDOT films wirelessly deposited in the absence of supporting electrolyte and those formed using a traditional three-electrode setup are indistinguishable. Together, these findings are important for the development of wirefree, multianalyte, detection devices, regioselective electrodeposition, and the production of advanced nano(bio)materials.

## Supporting Information Available

Diagram of the experimental setup and diagram of electromigration (PDF)
