# Supplementary material for: Electric Field Distribution in Bipolar Electrochemical Cells: Effects on the Wirefree Electrodeposition of Conducting Polymer Films
Source: Anal Chem. 2024 Dec 19;97(1):410–8. doi: 10.1021/acs.analchem.4c04454 (PMC11740180; doi:10.1021/acs.analchem.4c04454)
Supplement: Supplementary file 1 — ac4c04454_si_001.pdf [file ac4c04454_si_001.pdf]

Supporting Information

Electric Field Distribution in Bipolar Electrochemical Cells: Effects on the Wirefree Electrodeposition of Conducting Polymer Films

Áine Brady[a,b], Robert Forster [a,b]*

[a] National Centre for Sensor Research, School of Chemical Sciences,

Dublin City University, Dublin 9, Ireland, D09 V209

[b]FutureNeuro, SFI Research Centre for Chronic and Rare Neurological Diseases, Dublin City University, Dublin 9, Ireland, D09 V209

Corresponding Author: Robert Forster, School of Chemical Sciences,

Dublin City University, Dublin 9, Ireland, D09 V209. **Email:** Robert.Forster@dcu.ie

Table of Contents

Figure S1. Bipolar cell set up

Figure S2. Electromigration of charged oligomers. Scheme 1: Predominant migrational force across the BPE. Scheme 2: BP cell (feeder induced) migrational force.

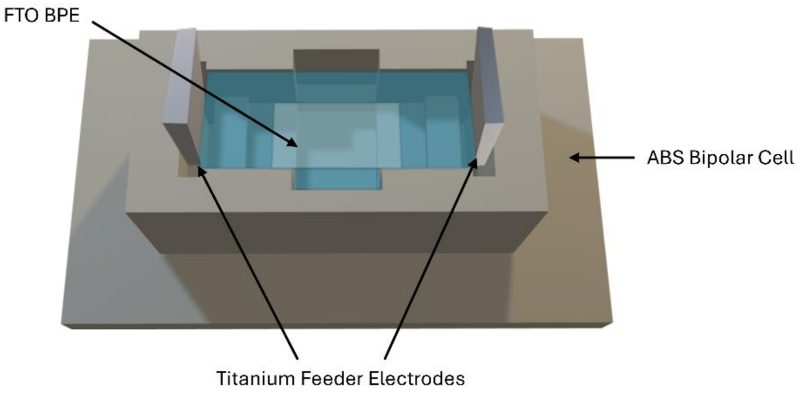

Figure S1. Bipolar cell set up

The dimensions and fabrication process of the bipolar cell are described in the experimental section of the manuscript.

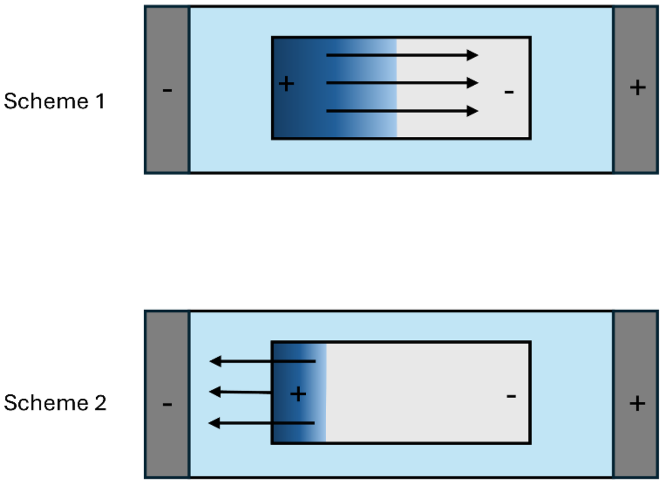

Figure S2. Electromigration of charged oligomers. Scheme 1: Predominant migrational force across the BPE. Scheme 2: BP cell (feeder induced) migrational force.
